# Subjective social status across the past, present, and future: status trajectories of older adults

**DOI:** 10.1007/s10433-024-00810-4

**Published:** 2024-05-23

**Authors:** Tim Kuball, Georg Jahn

**Affiliations:** Department of Psychology, Chemnitz University of Technology, Wilhelm-Raabe-Str. 43, 09120 Chemnitz, Germany

**Keywords:** Subjective social status, Aging anxiety, Well-being, Ageism, self-perception

## Abstract

Beyond objective indicators of social status (e.g., income or education), the subjective social status (SSS; i.e., the self-assessed position in a social hierarchy) is associated with psychological well-being and physiological functioning. Existing research has focused on older adults’ current status evaluations, neglecting perceived temporal stability or change in SSS which can further impact self-perception and emotional well-being. In the present study, we examined older adults’ (*N* = 191; mean age = 73.5) SSS with regard to their past, present, and future. Examining SSS for multiple time-points allowed us to identify profiles representing trajectories of status from the past to the future by conducting latent profile analysis. Furthermore, we tested associations of the identified trajectory-profiles with aging anxiety and negative affect. Results showed that, on average, participants anticipated higher future status losses than they had experienced in the past, regardless of age. In the more nuanced profile analysis, we identified four trajectory-profiles: A high (17%), a moderate (57%), and a low perceived social status (14%) trajectory, as well as a profile representing a perceived decrease in status (12%). While a lower status was associated with more aging anxiety and negative affect, most aging anxiety and negative affect was found for profiles representing a low initial status-level and a perceived decrease in status. Findings implicate that social status comparisons with others but also status comparisons with past- and future-selves are relevant for older adults. The discussion highlights the benefits of improving or stabilizing subjective assessments of status in later adulthood.

## Introduction

Social status is determined by respect and admiration as well as social influence and instrumental value to others (Anderson et al. 2015; Ridgeway 2001). Due to negative stereotypes about old age (Chan et al. 2012; Kleissner and Jahn 2020), older adults typically face a loss in social status attributed to them by others compared to younger people and compared to their younger selves. However, these age-related status expectations are not necessarily reflected in people’s subjective social status (SSS) evaluations and perceived temporal changes in SSS (Robertson and Weiss 2017). Although extensive research has been carried out on the SSS of older adults in the present moment, very little attention has been paid to the assessments of perceived past, present, and anticipated SSS. Unlike longitudinal studies, the assessment of multiple time points allows the investigation of individually perceived and expected change profiles and associated psychological outcomes relevant for health and well-being. Temporal comparisons with a past- or future-self offer reasonable and salient anchor-points to assess social changes and re-evaluate one’s perceived position in a social environment (de la Sablonnière et al. 2009). According to relative deprivation theory (Smith et al. 2012) not only comparisons with other people or other social groups, but also temporal comparisons within a single individual can result in affective reactions and individual behaviors related to health and well-being.

This study provides a first examination of older adults’ perceived and expected past, present, and future SSS. Beyond an averaged trajectory, we identified unique SSS profiles of individuals after retirement in a first step. These profiles allowed comparisons with earlier findings on age-based status attributions. Perceptions of temporal change or stability can impact self-perception and psychological well-being (Keyes 2000). We examined associations of the found trajectory-profiles with aging anxiety and negative affect as outcome variables.

## Facets of social status

Individuals high in status enjoy admiration by others, receive frequent positive social attention, and benefit from easier access to resources that can help them to accomplish personal goals (Anderson et al. 2015). Since status can be of material, social, and psychological value, individuals are motivated to obtain and maintain their status (Marr and Thau 2014; Pettit et al. 2010; Scheepers et al. 2009).

Most indicators of one’s SSS are grounded on social evaluations and comparisons and use the individual’s local social environment (community) or society as a reference. Typically, respondents are asked to indicate their subjective momentary position on a 10-rung status-ladder (Euteneuer et al. 2021; Hoebel et al. 2015). Beyond objective measures such as socioeconomic status (SES) based on financial success or education, high SSS has proven to be a reliable indicator for physical health-related quality of life (Euteneuer et al. 2021), life-satisfaction (Anderson et al. 2012), lower risk of functional decline (Chen et al. 2012), and better psychological functioning (Adler et al. 2000).

Alongside subjective and objective criteria, (perceived) age has been found to be a determinant of status attribution. Evidence shows that status ascribed to people of different age groups (age-based social status) can be described by an inverted U-shaped curve along the life-span (e.g., Baker 1985; Robertson and Weiss 2017). Middle-aged adults, holding the greatest societal resources, are ascribed the highest standing in a social hierarchy. The young and the old are ascribed a lower social standing in comparison. These age-based status attributions can be linked to negative portrayals of older adults that fuel ageist stereotypes (Robertson and Weiss 2017). Individuals are attributed a higher standing by others when they convey the impression of instrumental social value and might help to achieve own goals (Anderson et al. 2015)—a quality that might be denied to older individuals due to stereotypes portraying them as high in warmth and low in competence (Cuddy et al. 2005; Fiske 2017) or low in activity and openness (Chan et al. 2012). Everyday interactions in which being young is seen as good and being old is seen as bad, can negatively impact self-esteem and create a hostile environment for developing a healthy identity as an aging individual that includes mental representations of how one is viewed or valued by others (Gendron et al. 2020). Negative ageist stereotypes can be internalized and impact older adults’ implicit believes about aging and status (Levy 2009). In consequence, older adults try to distance themselves from their age group (Weiss and Lang 2012) and perceive other people of their age to have low social status but show more ambivalent believes about their personal social standing (Robertson and Weiss 2018). These implicit theories about development and aging may be reflected in processes of self-evaluation including individually perceived patterns of SSS across time.

## Subjective social status across time

Subjective status assessments may differ from age-based status ascribed by others. In longitudinal studies, older individuals (65–84 years) did not report a change in SSS when surveyed at two time points 10 years apart (Weiss and Kunzmann 2020) or even showed positive associations between age and SSS (Chen et al. 2012). In these studies, only mean values were considered, individual trajectories were not investigated further.

While longitudinal studies use data from multiple time points, subjective change can be assessed in the present time affording an examination of both, reflection on past experiences and anticipations, which can be represented in a variety of individual trajectories or change profiles that are lost when just considering data based on average trends (Röcke and Lachman 2008). Thinking back in time and reconstructing the past can be biased by implicit theories about development in certain age-related characteristics (McFarland et al. 1992; Ross 1989). Future self-views on the other hand can be shaped by internalized age stereotypes (Kornadt and Rothermund 2012) and future perspectives in older age can be linked to goal-relevant behaviors influential in health choices (Tasdemir-Ozdes et al. 2016). Younger people’s future perspectives are mainly defined by growth, while for older adults future time seems increasingly limited and their focus gradually shifts to a perspective of maintenance and loss avoidance (Ebner et al. 2006).

Past research defined individual profiles by change and stability across time (Goodman et al. 2015; Niu et al. 2021). Evidence suggests that individuals desire stability because it can satisfy a need for self-consistency (Keyes 2000). Niu et al. (2021) examined individual change profiles in SSS of college students and their associations with psychological well-being. Results revealed that young adults could be classified into profiles of high, mid, and low relative stability and an upward social mobility profile. Even though improvements in status can imply adaptive self-enhancements, the high stable profile was associated with the best outcomes in terms of psychological well-being. Perceived upward mobility in SSS, however, related to higher negative affect and was found to be harmful for the assessed well-being outcomes because it might imply financial or social stress.

Compared to younger individuals, older adults see less future opportunities to improve their social status (Weiss et al. 2022) and perceptions of age-related losses may be reflected in SSS trajectories (Blawert and Wurm 2021). When expecting decreasing status, intra- and interpersonal status comparisons can represent a mismatch between the intended and actual development which can activate a spectrum of affective reactions including anger, resentment, and worry (Bernstein and Crosby 1980; Brandtstädter 2007; Smith et al. 2012). Furthermore, subjective status evaluations can reflect how one feels to be seen or evaluated in relation to others. Not only how one sees themselves (personal), but also how one feels perceived by others (relational) is part of our identity as aging individuals. Both, personal and relational identities, are components of aging that can be fraught with anxiety if ageist stereotypes have been internalized (Gendron et al. 2020). To assess experienced negative affective reactions (e.g., discouragement, anger) as well as aging anxiety as a future-oriented emotion, we included respective outcome variables in the present study.

## The present study

According to relative deprivation theory, people compare themselves to other individuals but also to their past- and future-selves. However, research has mainly focused on present status evaluations, neglecting how older adults perceive the development of their SSS. We extended previous research and tested a new methodological approach in gerontology by employing five MacArthur-ladders for multiple time points instead of just one for present SSS, which allowed identifying profiles that are comprised of perceived past, present, and future SSS.

Drawing on previous research that examined change profiles (e.g., Niu et al. 2021; Röcke and Lachman 2008), we aimed to determine and distinguish stable profiles as well as profiles that represent a perceived change in status. Compared to younger people, older adults focus primarily on loss avoidance and perceive less opportunities for status improvement (Ebner et al. 2006; Weiss and Blöchl 2023). For this reason, we hypothesized that older people are likely to perceive a decline in their status, especially with respect to their anticipated future.

We further aimed at testing the association of the identified SSS-profiles to aging anxiety and affect that results from status comparisons. Losing status is negatively related to indicators of health and well-being (Anderson et al. 2012). Thus, we hypothesized that profiles that represent a lower subjective status are associated with heightened aging anxiety and more (justice-related) negative affect. Profiles representing a perceived decline in status should be associated with worse ratings compared to profiles representing stability.

## Methods

### Participants and procedure

We recruited for online participation via the newsletter distribution list of the Deutsche Seniorenliga e.V. and newspaper advertisements. In total, 206 older adults above the age of 65 completed the online survey. We excluded 15 participants from data analysis because they answered a control item incorrectly. The final sample consisted of 191 respondents in the range of 65–88 years of age (mean age = 73.5; SD = 5.75) including 125 men (65%). Overall, 52.9% of the sample indicated that they have a university degree or a degree from a university of applied sciences. The majority of the sample was retired (96%).

After giving informed consent, participants first conducted SSS ratings before the remaining scales were presented. Participation was voluntary and not compensated monetarily.

### Measures

#### Subjective social status across time

To assess self-perceived social status in the past, at present, and in the future, we employed an adjusted version of the MacArthur Scale of Subjective Social Status (Hoebel et al. 2015). Specifically, instead of just one ladder, participants were presented with five, ten-rung ladders placed next to each other for each of the five time points (10 years ago, 5 years ago, today, in 5 years, in 10 years). The instructions read as follows:

*“Please imagine a ladder with 10 rungs to show where people stand in Germany. By social status is meant prestige, social standing, or position in society. At the top(10), for example, are people with the most money, the highest education, and the best jobs. At the bottom(1) are those with the least money, the lowest education, and the worst jobs or no job. The higher you climb the ladder, the closer you are to the people at the top; the lower you are, the closer you are to the people at the bottom. Using the example of ladder-rungs, please indicate how you assess your social status in the past (10 years ago, 5 years ago), in the present, and in the future (in 5 years, in 10 years).”*

#### Aging anxiety

To measure participants’ aging anxiety, we used the relational ageism scale (Gendron et al. 2020). This scale is a refined version of the anxiety about aging scale by Lasher and Faulkender (1993) and incorporates subscales for internalized personal aging anxiety (e.g., „I expect to feel good about myself in my older age), relational aging anxiety (e.g., „People will see me as competent in my older age), and collective ageism (e.g., “I enjoy being around older people”) with Cronbach’s alpha of .73, .85, and .89, respectively. Participants indicated their agreement on a scale ranging from 1–*do not agree at all* to 7–*agree completely*. Items were presented in randomized order. Higher average scores indicate higher anxiety.

#### Negative affect

Five items were used to assess justice-related affect experienced in relation to disparities in status (*discouraged, angry, treated unfairly, content, optimistic*). Participants indicated their agreement on a scale ranging from 1–*not at all* to 7–*very much*. Items were presented in randomized order. Higher averaged scores indicate more negative feelings (Cronbach’s alpha = .79).

#### Subjective health

We assessed subjective health with a single item: “How would you describe your state of health in general?” answered on a scale ranging from 1–*very bad* to 7–*very good*.

#### Demographic information

Finally, participants were asked to indicate age, gender (0 = men, 1 = women, 2 = diverse), level of education (0 = no school degree—6 = university degree), and current work status.

### Statistical analysis

First, we examined descriptive statistics of all variables including mean status ratings to observe the average SSS trajectory across all participants. Bivariate correlations between the status ratings and demographic as well as outcome variables were calculated. To identify older adults’ past to future SSS trajectory-profiles, we conducted latent profile analysis (LPA) using Mplus (Version 8.10). We used only three timepoints (− 10 yrs., current, + 10 yrs.) to minimize global level-effects (Morin et al. 2016) of the separate SSS-ratings that might conceal potential shape differences^1^. We selected the best-fitting model based on multiple statistical fit values, namely Akaike Information Criterion (AIC), Bayesian Information Criterion (BIC), sample-size adjusted BIC (SABIC), Lo-Mendell-Rubin log-likelihood ratio test (LMR) and Entropy (indicator for accuracy of profile classification) as well as content-driven decisions (Spurk et al. 2020). BCH-procedure was used to test associations of profile-membership with aging anxiety, negative affect, and demographic variables that were defined as outcome variables. In this procedure, mean values of the outcomes are compared across the found profiles using the Wald Chi-squared test.

## Results

**Fig. 1:**
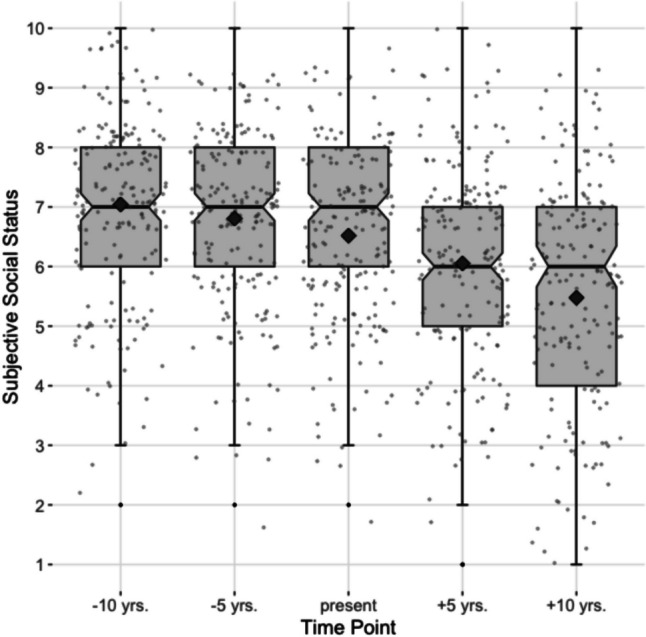
Averaged trajectory of subjective social status. *Note* The squares indicate the mean values

Analysis of descriptive statistics and the mean SSS trajectory indicated a perceived deterioration in status, especially for future time points (see Fig. 1). *T*-tests including mean status ratings for past (10 yrs. Ago—present; *t*(190) = 5.00, *p* < .001, *d* = .36) and future time points (present-10 yrs. from now; *t*(190) = 10.75, *p* < .001, *d* = .78) showed a significant decrease, with greater effects for the anticipated future.

Interestingly, correlational analysis showed that age was almost unrelated to status-level at all five time-points (with *r*s of − .03 to .06, all *p*s > .43), suggesting that, on average, a future decline in status is expected regardless of age. The outcome variables aging anxiety and negative affect were positively correlated (*r* = .42**, *p* < .001). See Table 1 for means, standard deviations, as well as correlations between SSS-ratings, demographics, and outcome variables.

**Table 1:** Correlations between status ratings for the five time points, demographics, aging anxiety, and negative affect

Variables	SSS − 10 yrs	SSS − 5 yrs	SSS	SSS + 5 yrs	SSS + 10 yrs
Age	.06	− .02	.02	− .03	− .03
Gender	− .06	− .10	− .05	− .04	− .06
Education	.20**	.17*	.13	.08	.05
Subjective health	.01	.12	.14	.14	.13
Aging anxiety	− .04	− .24**	− .41**	− .45**	− .42**
Negative affect	− .08	− .27**	− .40**	− .41**	− .40**
*M* (SD)	7.07 (1.58)	6.80 (1.49)	6.50 (1.51)	6.04 (1.73)	5.46 (2.04)

*N* = 191*;* *p < .05; **p < .01; Gender: 0 = men, 1 = women; Education: 0 = no school degree - university degree

### Trajectory-profiles of subjective social status

**Fig. 2:**
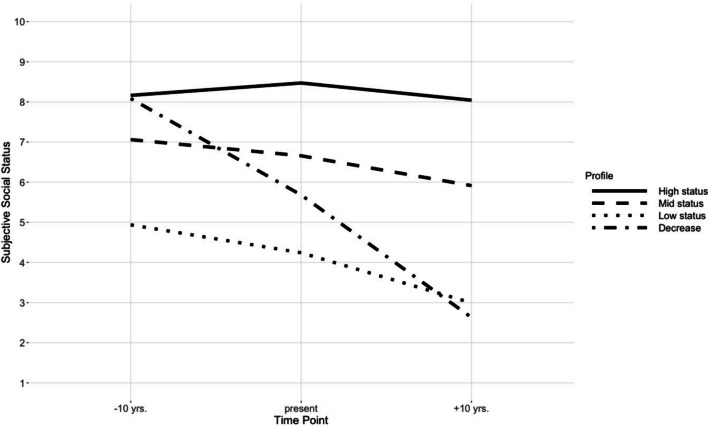
The four identified trajectory-profiles. *Note* The line plots present mean values of SSS for the participants assigned to the four profiles

As presented in Table 2, all reported fit statistics for the LPA models improved with an increasing number of profiles. Entropy values approaching 1 are ideal. The four-profile solution reached an entropy value of .82, which is considered a value that indicates a good separation between classes (Celeux and Soromenho 1996). Considering qualitative distinctions between the identified profiles, the three-profile solution produced profiles that differed only in their initial status-level. The four-profile solution however, produced an additional profile representing a decline in SSS from past to future. Increasing the number of profiles to five and six only resulted in more (stable) profiles that differed mainly in their initial status-level. Based on these content-driven considerations, the four-profile solution was determined to be the best fitting model. See Fig. 2 for the four identified trajectory-profiles.

**Table 2:** Statistical fit values of the latent profile analyses for the different profile solutions

No. of Profiles	LL	FP	AIC	BIC	SABIC	LMR(*p*)	Entropy
2	− 1055.96	10	2131.92	2164.44	2132.77	.002	.76
3	− 1029.73	14	2087.46	2132.99	2088.64	.10	.79
**4**	− **1010.28**	**18**	**2056.58**	**2115.12**	**2058.10**	**.18**	**.82**
5	− 994.38	22	2032.75	2104.30	2034.62	.32	.84
6	− 980.74	26	2013.47	2098.03	2015.67	.08	.88

Bold text indicates the selected profile solution

*N* = 191*;* *LL* Loglikelihood value (replicated in all solutions), *FP* Free parameters, *AIC* Akaike Information Criterion, *BIC* Bayesian Information Criterion, *SABIC* Sample-size adjusted BIC, *LMR(p)* *p*-value for the adjusted Lo–Mendell–Rubin-test

Profiles 1, 2, and 3 represented more stable profiles compared to profile four on a *high *(1), *mid *(2) and *low *(3) status-level. Profile 1 (17%) showed a slight increase in SSS for the past with a perceived decline for anticipated status. Profile 2 (57%) represented perceived stability in social status with only a small perceived decrease in status across the time-period in question. Compared to profiles 1 and 2, the anticipated decline in SSS was more pronounced for profile 3 (14%), suggesting a more negative perspective on future SSS developments for participants that indicated a lower initial status. 12% of all participants were classified in profile 4 (decrease) that showed a decline from past to future time points and thus indicated a more negative perspective on future SSS.

### Trajectory-profiles and outcome variables

Associations of profile-membership with the outcome and demographic variables were tested with the BCH-procedure. See Table 3 for means and standard deviations of the four profiles and results of the Wald tests. The four profiles showed no reliable differences with regard to age, gender, or subjective health (with *p* = .59, .53, and .45, respectively). With the Wald test remaining non-significant, only education of the included participants was reliably lower in profile 3 than profile 4 (*χ*^2^(3) = 5.93, *p* = .015). Supporting profile classification, aging anxiety and negative affect were more pronounced in profiles representing trajectories at lower initial status-levels. Profiles 3 (low status) and 4 (decrease) showed similar levels in aging anxiety and negative affect that were higher than for profiles 1 and 2. Profile 1 (high status) had the lowest mean aging anxiety and negative affect (superscripts in Table 3 indicate reliable pairwise differences).

**Table 3:** Equality tests of means across profiles using the BCH-procedure (Wald tests)

Outcomes *M* (SD)	Profile 1 High status (*n* = 32)	Profile 2 Mid status (*n* = 111)	Profile 3 Low status (*n* = 25)	Profile 4 Decrease (*n* = 23)	Wald
Age	74.42 (1.35)	72.92 (0.60)	74.01 (1.33)	74.36 (1.14)	*χ*^2^(3) = 1.91; *p* = .59
Gender	0.31 (0.10)	0.33 (0.50)	0.50 (0.11)	0.28 (0.12)	*χ*^2^(3) = 2.13; *p* = .53
Education	4.92 (0.32)	4.84 (0.15)^3^	4.01 (0.35)^24^	5.30 (0.33)^3^	*χ*^2^(3) = 6.50; *p* = .09
Subj. health	5.19 (0.28)	5.01 (0.13)	4.76 (0.27)	4.61 (0.36)	*χ*^2^(3) = 2.66; *p* = .45
Aging anxiety	2.79 (0.12)^234^	3.11 (0.08)^134^	3.67 (0.17)^12^	3.73 (0.23)^12^	*χ*^2^(3) = 29.80; *p* < .001
Negative affect	2.30 (0.22)^34^	2.55 (0.11)^34^	3.55 (0.28)^12^	3.47 (0.35)^12^	*χ*^2^(3) = 22.50; *p* < .001

Superscript digits indicate mean differences between designated profiles at *p* < .05

## Discussion

Our main goal was to explore older adults’ trajectories of SSS including past, present, and future perspectives. We identified unique status-profiles beyond an overall trajectory and examined their relation to aging anxiety and negative affect.

The overall SSS trajectory indicated that future declines were expected to be more pronounced than experienced past declines. Participant age, however, was not correlated with status-level for any of the five time points and was not associated to profile membership. Exploring groupings of individual status trajectories, we identified four distinct profiles: Three profiles were characterized by a high, moderate, and low initial status-level. The fourth profile showed a steeper decline in status from past to future time points. All profiles revealed a more negative perspective on future compared to past status developments. Profiles representing higher social status and more temporal stability in SSS were associated with less aging anxiety and negative affect. Least anxiety toward aging and negative affect was found in participants categorized to profile 1 (high status). Corroborating our assumption that a perceived decrease in status is associated with more negative affect and anxiety toward aging, profiles 3 and 4 were associated with the highest values on these variables.

Overall, predictions of own social status in the future were negative compared to experienced past trajectories, regardless of chronologic age. This also accords with earlier observations, which showed that older adults compared to younger people perceive social hierarchies as less malleable due to diminishing future opportunities to move up the status-ladder (Ebner et al. 2006; Weiss and Blöchl 2023). As mentioned in the literature review, perceived decreases in SSS may reflect negative future self-views (Kornadt and Rothermund 2012) and age-based status beliefs of declining social status in older adulthood with a lower likelihood of improvements in social status for the personal future (Robertson and Weiss 2017). Other research indicates a positive association between SSS and a countries’ modernization index. This relation was further influenced by the employment rate among older adults in the country under study (Vauclair et al. 2015). Hence, perceptions of future status loss might also result from anticipated societal, demographic, or cultural changes affecting older age groups.

Participant age was unrelated to status-level. While this result is consistent with longitudinal findings on SSS in earlier studies (Weiss and Kunzmann 2020; Chen et al. 2012), the method of using status-ladders for multiple time points in the present study revealed perceived and expected within-person changes in subjective status for a subset of participants. That is, the methodological approach of assessing multiple time points at once, may reinforce direct comparisons with past- and future-selves. Comparisons with past-selves may have encouraged older adults to reflect on earlier times (e.g., before retirement). The anticipation of future status development can prompt future self-views characterized by age-related losses (Kornadt and Rothermund 2012).

The more nuanced analysis of trajectory-profiles showed that more than half of all participants perceived their status to remain relatively stable with increasing age. Participants that indicated perceived status-stability on a moderate level constituted the largest group. Perceived stability in SSS may indicate that the individual has managed to respond consciously or unconsciously to adaptive challenges of aging by adjusting personal goals to situational constraints and personal resources or by altering external circumstances to match personal interests (Brandtstädter 2007). Research on subjective well-being and satisfaction shows that positive outcomes arise not directly from good life conditions, rather from judgments that characterize a life situation as good in relation to a specific standard of comparison (Tesch-Römer and Wurm 2006). Similarly, social comparison standards indicative of one’s social status might be adjusted in a self-stabilizing manner (Greve and Wentura 2010).

12% of the participants were classified to profile 4 that indicated a perceived decrease in status compared to a past-self and an expected decrease in status for the future. These recollections and expectations can be biased by an individual’s implicit theories about aging. That is, for qualities and traits theorized to decline with age, older adults tend to remember having these traits more pronounced when they were at a younger age (McFarland et al. 1992). Since age-based social status is expected to decline with age, older participants might be biased about their past social standing and rated their retrospective status higher than their current status. Another possible explanation for the found results are objective changes in the older adults’ reality of life that have led to the stated changes. Previous results on middle-aged adults suggest that an individuals’ evaluation of SSS is a process that “appears primarily to involve cognitive averaging of standard markers of socioeconomic position” (Singh-Manoux et al. 2003, *p.* 1331) such as income, occupational position, and feelings of financial security. Thus, in our sample, the loss of a professional position and retirement may have contributed to the perceived decrease in subjective status. Furthermore, experiences of ageist discrimination can impact perceptions of aging (Han and Richardson 2015) and result in feelings of being devalued as a person with increasing chronological age (Boudjemadi et al. 2017).

In contrast to earlier findings that focused on young adults (Niu et al. 2021), no profile representing upward mobility was identified. Lifelong development involves periods of stability that are characterized by a focus on maintenance but also periods characterized by growth or losses. Growth requires biological plasticity and a social environment that provides access to resources that enable skill acquisition and improvement in overall functioning. These resources are primarily available to younger adults. With more limited resources at older ages, the primary focus is shifting to maintaining and avoiding loss of functioning (Baltes 1987; Ebner et al. 2006).

Giving support to our hypotheses in relation to aging anxiety and negative affect, our findings showed that participants assigned to profiles representing a status trajectory on a low level or a perceived decrease in status, reported the highest levels on the outcome variables. Negative affective reactions such as anger and resentment are considered key correlates in relative deprivation theory (Smith et al. 2012). The appraisal of interpersonal comparisons can trigger negative justice-related affect if the person views the differences as unfair and feels they deserve better, believes that the experienced deprivation is illegitimate, unlikely to change, and not within their own responsibility (e.g., Crosby 1976; Ellemers 2001; Smith et al. 2012). Participants categorized to profile 3 or 4 might feel they deserve a higher status but are denied this position due to their age. Particularly, when older individuals realize that they will not be able to regain their previous social status, feelings of deprivation can arise (Crosby 1976). Feeling unable to become the person one aspires or aspired to be can serve as both, a crisis or a turning point in personal development. Depending on personal tendencies of self-regulation, anger or worry can change into hope and happiness, or into feelings of hopelessness and despair (Brandtstädter 2007).

Beyond affective reactions resulting from disadvantageous comparisons, aging anxiety encompasses how one feels about themselves and how one feels evaluated by others as an aging individual (Gendron et al. 2018, 2020). With regard to the present study, individuals who reported high levels of aging anxiety may have internalized more negative ageist beliefs (Levy 2009) and perceive status-relevant personal and relational aspects as declining with age. More precisely, increased aging anxiety might be associated with perceived or anticipated age-related losses in social, cognitive, or physical domains relevant to maintaining status-relevant attributes such as personal autonomy and cognitive health.

Our findings suggest that the desire to maintain social status persists into later adulthood. Older individuals compare themselves not only with members of the same (age) group but also to members of other (in this case younger) age groups and with past- and future-selves. Relative comparisons have received little attention in aging research. Consistent with relative deprivation theory (Smith et al. 2012), present results showed that social comparisons including a more pessimistic outlook on one’s future, related to negative affective states in the present and anxieties about future aging in older adults.

### Limitations and future directions

We assessed data about past, present, and future SSS at a single point in time. Due to the cross-sectional nature of the data, findings connected to SSS trajectories can be confounded by various factors (Lindenberger et al. 2011). As mentioned above, societal factors such as employment rate among older adults (Vauclair et al. 2015) can influence SSS. More longitudinal studies are needed to observe the causal link of dynamic changes in SSS and its impact on factors related to health and emotional well-being.

Our sample had a high level of education. Hence, found status trajectories may not be representative for people of all educational backgrounds. Only participants over the age of 65 were included, most of whom were already retired. Evaluations of past status changes might be influenced by specific events such as retirement. However, our results did not indicate changes in SSS among the youngest participants in our sample that may have resulted from entering older adulthood or retirement. In a similar vein, the older participants of our sample did not report lower future status than the younger participants and it remained uncertain how the prospect of death might influence a future status assessment. In the current study, we focused on a period of 20 years to indicate perceptions of status change. A finer differentiation or an extended time perspective could reveal further interesting perceived changes in SSS. Future studies could also delve deeper into cultural or societal factors (e.g., modernization or demographic change) that could influence status perceptions in a positive or negative way.

The MacArthur scale is focusing on socioeconomic factors and uses society as a benchmark for social status (Hoebel et al. 2015). In a meta-analysis (Zell et al. 2018), high correlations were found between community and society as reference groups for status assessments. Yet, in older populations, mental health associations with SSS were more pronounced using the local community as a reference. Extending on this, Euteneuer et al. (2021) found that only local community SSS predicted mental health-related quality of life in a longitudinal study. Focusing on the closer, more benevolent social environment or community as a status reference could be an adaptive strategy to stabilize current status perceptions into old age. Moreover, by focusing on socioeconomic factors, older people may feel systematically disadvantaged compared to other age groups. With increasing age, definitions of status-relevant attributes may shift toward factors like cognitive functioning, physical health, or social engagement. A broader definition of social status could produce stronger associations with variables indicative of well-being. While the MacArthur scale aids comparability in SSS research, further research is needed to explore age-related changes in status definitions and the influence of proximal or distal reference groups on SSS stability or change.

We used latent profile analysis to extract trajectory-profiles. A rule of thumb from past research assumes a sample size of 500 to accurately identify profiles. Furthermore, profile classification was based on multiple social status ratings that were correlated with each other. This might have impacted qualitative differences in the found trajectories (Morin et al. 2016). Although our sample size is smaller, distribution of the included cases and accuracy of profile classification was in line with literature on best practice (Spurk et al. 2020).

### Implications

In the present paper, we tested a new methodological approach for gerontology, combining SSS evaluations for multiple time points. Examining the dynamic temporal changes of age-related status that aging individuals experience helps to understand and address anxieties and affective reactions connected to aging. Regardless of an individual’s definition, status reflects perceived recognition and appreciation. Our findings indicate that dynamic changes in SSS continue into old age and are related to affect and concerns associated with aging.

Our findings have implications at the individual-level and at the societal-level. The belief to maintain status or perceiving opportunities to move up the status-ladder in the future can buffer adverse effects of subjective status loss on psychological well-being (Weiss and Blöchl 2023). Stereotypes that negatively portray aging as a process of decline and reduced social standing should be addressed to improve the perspective on aging. Status beliefs and underlying ageist stereotypes can be improved in a variety of ways: Higher employment among older adults (Vauclair et al. 2015) and positive older role models (Robertson and Weiss 2017) can help to convey images of high status in older age. While individual pathways to retirement should be considered, continuing productive activity after retirement (e.g., voluntary work) can be perceived as fulfilling and creates value for society at the same time (Wanka 2020). Older individuals should be increasingly enabled and motivated to pass on their knowledge and skills to younger generations. Creating opportunities for intergenerational exchange helps challenging negative stereotypes on both ends (Cortellesi and Kernan 2016; Kuball et al. 2023) promotes generativity and intergenerational learning (Pratt 2013), and might reduce perceived differences in SSS.

As life expectancy increases, it is even more relevant to counteract exaggerated anxieties and negative feelings about old age. The present findings confirm that the perception of a higher and more stable social status is linked to a better outlook on aging. Measures that help to better integrate older people and make them feel valued as members of society can contribute to well-being in aging societies.

## Data Availability

The data supporting the findings of this study is available at: https://osf.io/3tgbz/?view_only=eeb5a63831ad436896534fab4da333d2.

## Footnotes

1 No qualitative differences compared to using all five time points emerged. Using only three time points however, produced a better distribution across the four profiles.
